# Five grand challenges for decarbonization of China's energy system

**DOI:** 10.1093/nsr/nwaf035

**Published:** 2025-02-17

**Authors:** Zhijun Jin, Chuan Zhang

**Affiliations:** Institute of Energy, Peking University, China; Institute of Energy, Peking University, China

Decarbonization of energy system is the key for achieving China's carbon neutrality, yet the success of China's energy transition is a complicated issue that depends on many interdisciplinary challenges. Herein we highlight five most important ones among such challenges.

*Optimal energy transition pathway.* There is more than one pathway to achieving a net-zero-emission energy system. Each pathway features its own emission peaking amount, pace of emission reduction after carbon peak, and most importantly the final energy mix in a carbon constrained world. Interestingly, such an energy mix varies a lot among different studies. Taking coal consumption for net-zero power generation as an example, this ranges from 0.1 Gton to 1 Gton, which presents huge uncertainty for related capacity planning and decision making. In the real world, it is hard to judge which pathway is better because the energy system is a complex system involving different aspects of the economy, society, resources, technology, etc. How to evaluate the multidimensional impact and feasibility of different energy transition pathways constitutes the very first open question, requiring shared wisdom from academia, industry and government together.

*Decarbonization versus Defossilisation.* When it comes to the decarbonization of the energy system, fossil fuel remains the elephant in the room. Burning or refining fossil fuel, including coal, oil and gas, for fuel and chemicals still accounts for the bulk of China's energy landscape. There is no doubt that unabated fossil fuel use has no room in a carbon neutral energy system, yet when and how this use can be phased out remains unclear. More fundamentally, do we need decarbonization or defossilization? Fully divorcing from fossil fuel seems impossible as long as we still need carbon-based polymers and chemicals for our basic needs; in this sense, material use of fossil fuels will definitely stay. Even though several studies have proposed a 100% renewable energy system, criticisms about such a pathway are also evident. To better understand this debate, further research is needed.

*Green premium.* Green premium is the term that Bill Gates used to describe the additional cost of a low-carbon product or service over its counterpart that emits more carbon. Behind such a green premium is the fact that current renewable-based electricity, heat and fuel production routes have no cost advantage compared to their fossil competitors. While technological innovation and the learning effect could bring down the cost of emerging green technologies (the cost evolution of solar photovoltaics is a well-known example of this), the urgency for climate action requires the future acceleration of such innovations. In the end, pricing carbon will unavoidably reallocate the cost and benefits among government, companies and consumers. To design an efficient system for such economic needs will require novel interventions.

*Hydrogen economy.* Hydrogen, together with its derivates such as ammonia and methanol, has attracted a lot of attention during the current energy transition. But hydrogen is nothing new to the energy community. More than 30 million tons of hydrogen were produced in China in 2023, mainly for the chemical industry. The production of this hydrogen is mainly via coal gasification, which is assigned the color grey for its high carbon intensity. Production of green hydrogen through green electricity offers a promising route for variable renewable energy accommodation, using hydrogen for multiple end users. Yet the success of the hydrogen economy relies on there being a localized, stable supply and demand all year round. With regard to storage and transportation, more concerns have to be addressed.

*Carbon capture, utilization and storage.* Carbon capture, utilization and storage, known as CCUS, refers to the integrated process of CO_2_ capture, transport, utilization and storage to prevent CO_2_ release into the atmosphere. The role of CCUS in a future net-zero-emission energy system is two-sided: on the one hand, it could have a unique role in reducing the carbon intensity of fossil-fuel-fired power and industrial systems; on the other hand, it must be noted that the rhetoric on CCUS has not been turned into a reality so far. Even though most CCUS technologies have already reached a commercial level, upscaling of CCUS from megaton to gigaton to produce material climate change mitigation effects still lacks enough incentive. Setting up the required storage potential, raising the required investment and laying out a source-sink matching infrastructure would accelerate such CCUS industrial applications and increase our understanding of CCUS as a major climate mitigation option.

To conclude, achieving China's carbon neutrality without a fundamental transition of energy system seems inconceivable. Yet the question of how to transform the world’s most complicated energy system into a reliable, efficient and clean one has not been answered yet, and more discussions on such topics are called for.

